# Observed increases in North Atlantic tropical cyclone peak intensification rates

**DOI:** 10.1038/s41598-023-42669-y

**Published:** 2023-10-19

**Authors:** Andra J. Garner

**Affiliations:** Department of Environmental Science, Rowan University, Glassboro, NJ 08028 USA

**Keywords:** Atmospheric science, Climate change, Climate-change impacts

## Abstract

Quickly intensifying tropical cyclones (TCs) are exceptionally hazardous for Atlantic coastlines. An analysis of observed maximum changes in wind speed for Atlantic TCs from 1971 to 2020 indicates that TC intensification rates have already changed as anthropogenic greenhouse gas emissions have warmed the planet and oceans. Mean maximum TC intensification rates are up to 28.7% greater in a modern era (2001–2020) compared to a historical era (1971–1990). In the modern era, it is about as likely for TCs to intensify by at least 50 kts in 24 h, and more likely for TCs to intensify by at least 20 kts within 24 h than it was for TCs to intensify by these amounts in 36 h in the historical era. Finally, the number of TCs that intensify from a Category 1 hurricane (or weaker) into a major hurricane within 36 h has more than doubled in the modern era relative to the historical era. Significance tests suggest that it would have been statistically impossible to observe the number of TCs that intensified in this way during the modern era if rates of intensification had not changed from the historical era.

## Introduction

Tropical cyclones (TCs) are the most damaging natural hazard to regularly impact the U.S. Atlantic and Gulf coasts^1–4^. From 2012 to 2022, over 160 “billion-dollar” weather and climate disasters impacted the U.S; 24 of these events were TCs, including the six costliest disasters on record during this time^5^. Many of the most damaging TCs to impact the U.S. in recent years have been notable for the speed at which they have intensified. For instance, Hurricane Maria (2017), the climate disaster with the highest death toll since 1980, and the 4th highest economic cost in the last four decades, strengthened from a tropical storm to a Category 5 hurricane on the Saffir-Simpson scale in just over 48 hours^5–7^. Hurricanes Harvey (2017), Ian (2022), Sandy (2012), Ida (2021), and Irma (2017), the five other costliest U.S. weather and climate disasters in the last decade, all similarly strengthened rapidly, with most evolving from tropical storms to major hurricanes (Category 3 on the Saffir-Simpson scale or greater) in under three days^5,8–12^.

The fastest TC intensification rates often occur in areas of unusually warm upper ocean and sea surface temperatures (SSTs)^13–16^. These warm waters serve as a critical energy source for the strengthening storms which act as heat engines, transporting excess warmth from the oceans and atmosphere in the tropics to higher latitudes^17^. As anthropogenic emissions have warmed the planet, the world's oceans have warmed at the surface, where average temperatures have increased ~ 0.88 °C from 1850–1900 to 2011–2020^18^. The rate at which ocean surfaces have warmed has also accelerated, with 0.60 °C of this warming occurring since 1980^18^. Considering the role of warm upper ocean water and SSTs in the fastest TC intensification rates^13–16^, it is reasonable to expect that we may observe an increase in TC intensification rates that coincides with warming ocean temperatures in recent decades^19–24^. Given the highly-damaging nature of many TCs that intensify rapidly, and the operational and forecasting challenges posed by TCs that intensify most quickly^25,26^, there is an urgent need to better understand how intensification rates of TCs may already have changed in a warming climate.

Various past studies have sought to understand how rapid intensification of TCs may evolve in a warmer climate, including work focused on understanding how intensification events that occur within certain regions or fall above a certain threshold may change over time^22,27–34^. For example, Ref.^33^ examined intensification trends in landfalling TCs in East and Southeast Asia, and found that a 12–15% increase in the intensity of such storms at landfall was primarily due to an increase in the rate at which they intensified. In the Atlantic basin, multiple studies have sought to understand how TC intensification rates have changed near major coastlines. For instance, Ref.^22^ found that there was an increased likelihood for TCs to intensify quickly near the U.S. coast during times when basin-wide conditions were generally less favorable for such intensification events. Also focusing on intensification near U.S. coastlines, Ref.^28^ found that although the mean 24-h intensification rate of TCs increased by 1.2 kts/6 h near the U.S. Atlantic coast from 1979 to 2018, no similar increase was observed along the U.S. Gulf coast. Using a similar focus region, Ref.^32^ found that, when considering U.S. landfalling TCs, there was a tendency for TCs that intensified rapidly in the 24 h prior to landfall to decay more slowly after landfall.

Elsewhere in the Atlantic, Ref.^27^ notes that in the central and eastern tropical Atlantic, the 95th percentile of 24-h TC intensity changes increased at 3.8 kts per decade from 1986 to 2015. Using the same threshold of the 95th percentile of 24-h intensity changes to define rapid intensification, Ref.^30^ suggest that it is possible to detect an anthropogenic-related increase in Atlantic TC rapid intensification rates. Adopting a slightly different threshold of 30 kts/24 h to define rapid intensification, Ref.^29^ identify no trend in TC rapid intensification tied to warming from 1950 to 2014, but do note key spatial and temporal patterns in rapid intensification events in the Atlantic.

This study adds to the considerable previous research efforts described above by developing a broader assessment of overall basin-wide changes in the magnitude of peak Atlantic TC intensification rates. This work focuses on the Atlantic basin as a whole, rather than a subset of storms that occur in a specific portion of the basin. Furthermore, no arbitrary thresholds of intensification are used in this study to classify a TC rapid intensification event—instead, the work fills a key knowledge gap by assessing overall changes to the peak intensification rates achieved by all TCs across 12-, 24- and 36-h windows during the 5 decades spanning from 1971 to 2020. Results indicate broad increases to observed TC intensification rates over the past 50 years. These findings illustrate a vital need to not only work towards climate mitigation to limit future warming and thus additional changes in TC intensification rates, but also for emergency preparedness plans and resilience measures that will allow our coastlines to adapt to TCs that have already begun to exhibit increased rates of strengthening.

## Results

Changes to the observed maximum intensification rates of TCs from the Atlantic hurricane best track database (HURDAT2)^35^ are considered for a historical era (1971–1990), an intermediate era (1986–2005), and a modern era (2001–2020). The intermediate era contains five years of data that overlap with each of the historical and modern eras. Furthermore, the intermediate era ends in 2005, after which the Stepped Frequency Microwave Radiometer was installed on all National Oceanic and Atmospheric Administration hurricane research aircraft to help improve measurements and data collection related to maximum TC wind speeds^36^. The intermediate era is therefore useful for illustrating the temporal progression of TC intensification rates across the past 50 years, including across time periods that rely upon various instrumentation and data collection methods.

Maximum intensification rates are calculated for 12-h, 24-h, and 36-h windows along each track. The maximum intensification value of each TC for a particular window is defined as the greatest increase in wind speed across any window of that length during the lifetime of the storm (see “Methods” section). A variety of statistical analyses are used to assess how the maximum intensification rates of TCs across 12-, 24-, and 36-h windows have evolved over time. Results show significant changes to maximum TC intensification rates from the historical to the modern era.

### Increasing magnitudes of peak tropical cyclone intensification rates

**Figure 1:**
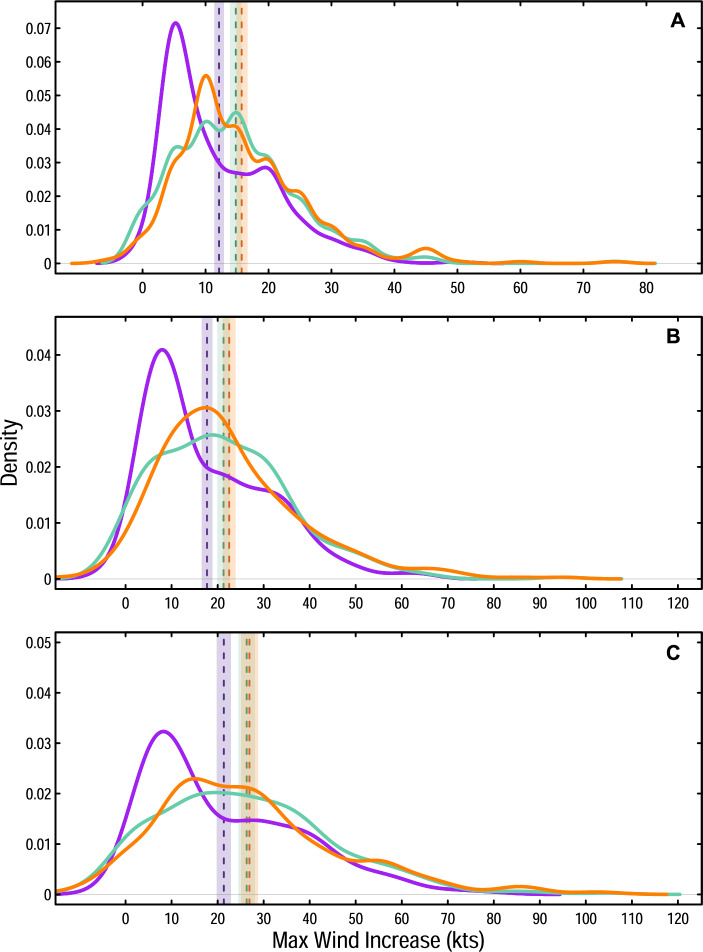
Probability density functions of tropical cyclone intensification rates. Distribution of tropical cyclone intensification rates for the historical era (1971–1990; purple), intermediate era (1986–2005; teal), and modern era (2001–2020; orange). Distributions of peak intensification rates are shown for (**A**) 12-h windows, (**B**) 24-h windows, and (**C**) 36-h windows. The mean for each distribution is shown by a dashed line, with shading to show the 90% credible interval surrounding the mean.

Distributions of observed maximum intensification rates across 12-, 24-, and 36-h windows show increasing intensification rates from the historical to the modern era (Fig. 1). The mean maximum intensification rates for a 12-h window increase by ~ 28.7% from the historical era (12.2 kts) to the modern era (15.7 kts). Similarly, mean maximum intensification rates across 24-h windows grow by ~ 27.1% from the historical era (17.7 kts) to the modern era (22.5 kts), and mean maximum intensification rates across 36-h windows increase by ~ 26.3% from the historical era (21.3 kts) to the modern era (26.9 kts). These increases are significant at a 90% credible interval (Fig. 1). Though intermediate and modern mean maximum intensification rates are not statistically significantly different from one another for any time window, all windows exhibit monotonic increases in mean maximum intensification rates across the historical, intermediate, and modern eras (Fig. 1).

**Figure 2:**
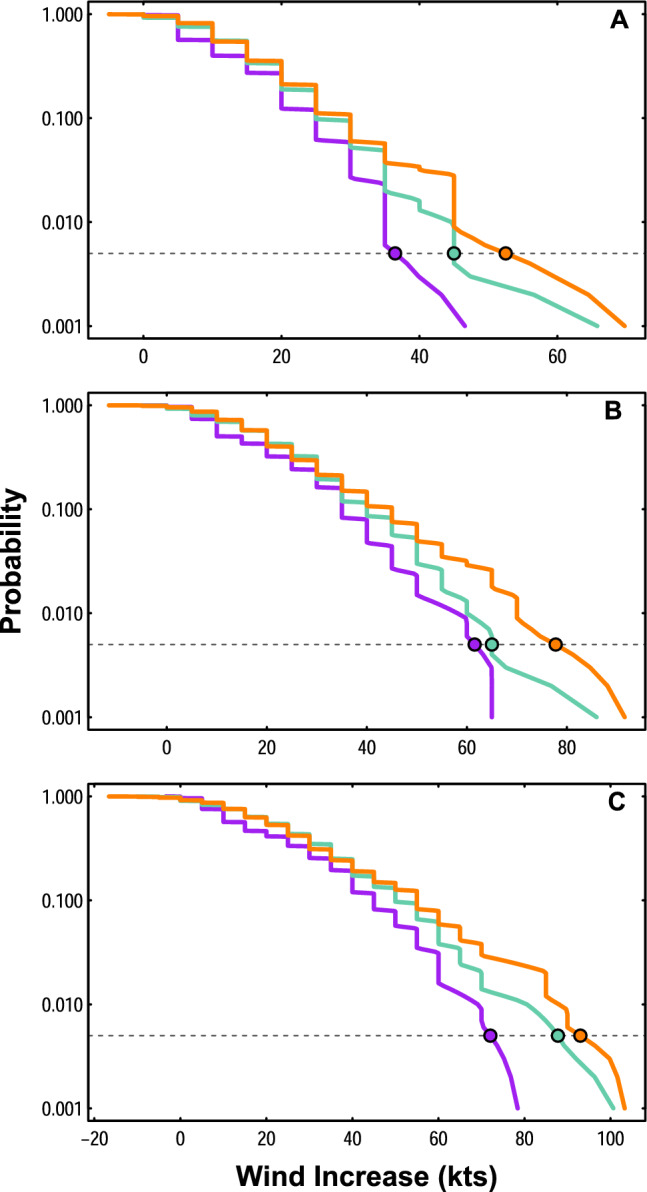
Survival functions of tropical cyclone maximum intensification rates. Survival functions showing probabilities of lifetime maximum intensification rate of TCs during (**A**) 12-h windows, (**B**) 24-h windows, and (**C**) 36-h windows for the historical era (purple), intermediate era (teal) and modern era (orange). Dashed lines and colored points indicate the 99.5th percentile for all plots and eras.

Increases in mean maximum intensification rates are accompanied by correspondingly larger increases to more extreme maximum intensification rates. For example, the 99.5th percentile of maximum TC intensification rates during a 12-h window expands from 36.5 kts (historical) to 45.0 kts (intermediate) to 52.5 kts (modern; Fig. 2a). Observations illustrate similar increases to the 99.5th percentile of maximum intensification rates for a 24-h window (historical: 61.6 kts; intermediate: 65.0 kts; modern: 77.8 kts; Fig. 2b) and a 36-h window (historical: 72.1 kts; intermediate: 87.8 kts; modern: 93.0 kts; Fig. 2c).

**Figure 3:**
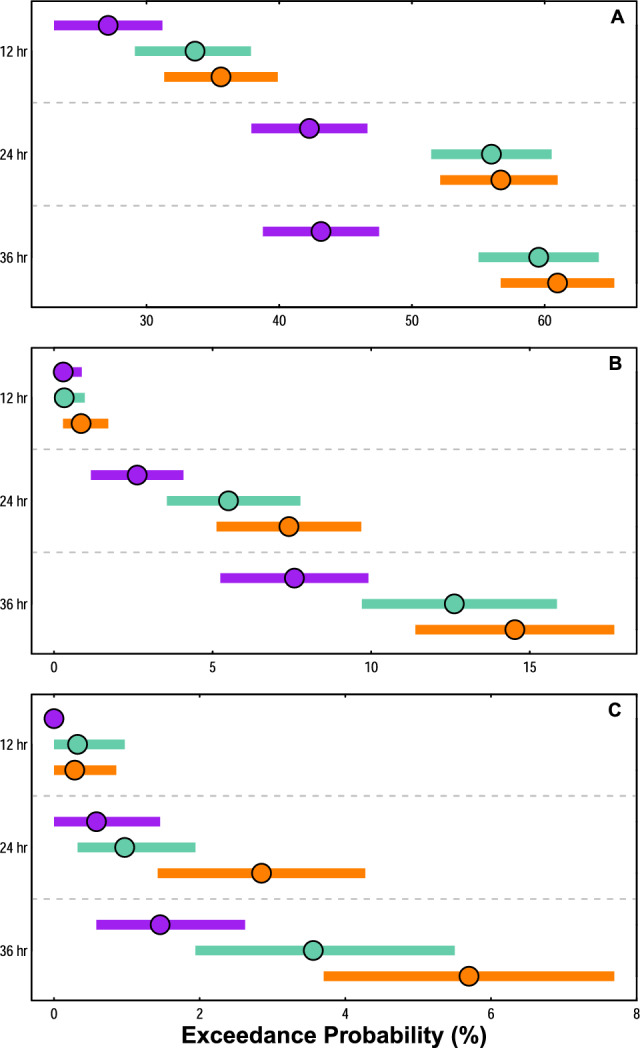
Exceedance probabilities of tropical cyclone maximum intensification rates. Probabilities of TCs exceeding an intensification rate of (**A**) 20 kts, (**B**) 50 kts, and (**C**) 65 kts across 12-h, 24-h, or 36-h windows for the historical era (purple), intermediate era (teal), or modern era (orange). Circles represent the observed exceedance probabilities; colored segments show the bootstrapped 90% credible interval of the observed exceedance probability.

It also becomes more likely for TCs to exceed particular intensification rates during 12-, 24-, and 36-h windows over time (Fig. 3). For example, the probability of a maximum intensification rate during a 12-h window either meeting or exceeding 20 kts grows from 27.1% in the historical era to 35.6% in the modern era—an increase that is significant at a 90% credible interval (Fig. 3a). Similarly significant increases in the likelihood of a 20-kt or greater maximum intensification rate occur from the historical (42.3%) to the modern era (56.7%) for a 24-h window, and from the historical (43.2%) to the modern era (61.0%) for a 36-h window (Fig. 3a). Furthermore, it is significantly more likely for an intensification of 20 kts or more to occur within just 24 h in both the intermediate and modern eras than it was for such an intensification to occur within 36 h during the historical era (Fig. 3a).

Peak intensification rates that either meet or exceed 50 kts also become more likely over time. For instance, the probability of maximum intensification rates meeting or exceeding 50 kts within 24 h grows from 2.6% during the historical era to 7.4% in the modern era (becoming ~ 2.8 times more likely), while the probability of such an intensification rate occurring within 36 h grows from 7.6% in the historical era to 14.5% in the modern era (becoming ~ 1.9 times more likely). Both of these increases are significant for a 90% credible interval (Fig. 3b). By the modern era, it becomes about as likely for a 50-kt (or greater) wind increase to occur within 24 h as it was for this type of wind increase to occur within 36 h during the historical era.

Though 65-kt intensification rates occur far less often, there are nonetheless significant increases (90% credible interval) in the chance of such intensification happening within both 24 h (2.9% chance in the modern era compared 0.6% chance in the historical era) and 36 h (5.7% chance in the modern era compared to 1.5% chance the historical era; Fig. 3c). These findings indicate that in the modern era, it is ~ 4.9 times more likely for a 65-kt intensification to occur within 24 h, and ~ 3.9 times more likely for such an intensification to occur within 36 h than it was for such intensification rates to occur during the historical era.

### Impact of changing intensification rates on Saffir–Simpson classifications

**Figure 4:**
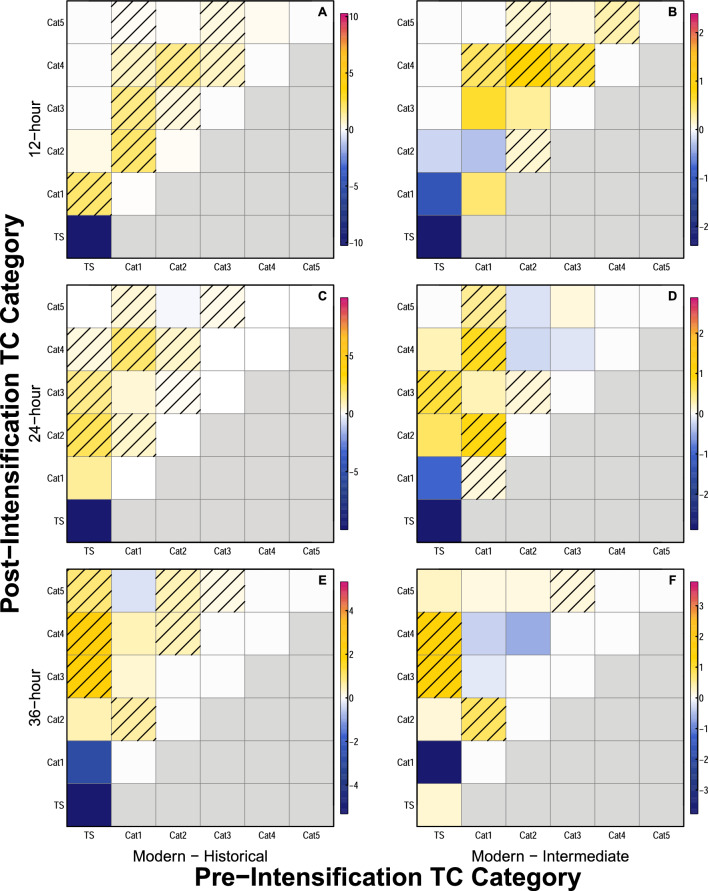
Heatmap of differences in tropical cyclone Saffir–Simpson category changes. Heatmaps showing differences in the percentage of TCs that fall into specific pre- and post-intensification TC categories between the modern and historical eras (**A,C,E**), and modern and intermediate eras (**B,D,F**). Differences are shown for peak intensification events across (**A,B**) 12-h windows, (**C,D**) 24-h windows, and (**E,F**) 36-h windows. Shading indicates grid cells for which the number of intensification events in the modern era would have had ≤ 5% chance of occurring in the time period used for comparison, based on analyses using a binomial cumulative distribution function. Units for colorbars are percentages. Note that because the focus of the analyses presented here is on intensification, blocks that would indicate weakening are gray.

Increases in peak intensification rates affect the time required for TCs to transition from one category to another on the Saffir–Simpson scale^7^. A comparison of the percentage of peak TC intensification events that fall into various pre-intensification and post-intensification TC categories suggests that modern TCs are more than twice as likely to intensify from a Category 1 hurricane or tropical storm (hereafter, “weak TC”) into a major hurricane (Category 3 or greater; hereafter, “major TC”) within either 24 or 36 h compared to their historical counterparts (Fig. 4, Fig. S1). Specifically, for a 24-h window, 8.12% of peak intensification events result in TCs intensifying from a weak TC into a major TC in the modern era, compared to 3.23% of peak intensification events that cause TCs to intensify in the same way in the historical era. For a 36-h window, 10.3% of peak intensification events result in modern TCs intensifying from a weak TC into a major TC, while only 4.23% peak intensification events cause TCs evolve in the same way during the historical era. For a 12-h window, modern TCs are more than three times as likely as historical TCs to undergo such an increase in TC category, with 3.58% peak intensification events resulting in TCs evolving from weak TCs into major TCs in the modern era, but only 1.06% of peak intensification events causing TCs to evolve this way during the historical era (Fig. 4, Fig. S1).

Although not normalized to account for varying storm frequency during different eras, the combined totals of peak intensification events that result in TCs intensifying from weak TCs into major TCs during each era suggest similar increases over time (Table 1). No tropical storms transition to a major TC during any era within a 12-h window, but there are substantial increases in the number of peak intensification events that cause TCs initially classified as Category 1 to intensify into major TCs within 12 h from the historical era (9 events) to the intermediate era (18 events) to the modern era (28 events; Table 1, Fig. 4). For a 24-h window, there are increases in both the number of peak intensification events that cause tropical storms to become major TCs (3 historical, 9 intermediate, 17 modern) and the number of peak intensification events that cause Category 1 TCs to become major TCs (21 historical, 30 intermediate, 43 modern; Table 1, Fig. 4). This trend is similar for the number of peak intensification events that result in tropical storms becoming major TCs within 36 h (13 historical, 27 intermediate, 49 modern). Variations in the number of peak intensification events that cause Category 1 TCs to become major TCs within 36 h are less consistent over time (16 historical, 21 intermediate, 19 modern; Table 1, Fig. 4), however the smaller number of modern storms here may be partially driven by the very large number of modern intensification events (49) that cause TCs to intensify directly from tropical storms to major TCs within 36 h (Table 1).

**Table 1:** Total intensification events that result in a TC evolving from a tropical storm to a major hurricane, or from a Category 1 hurricane to a major hurricane over all window lengths during each era.

Window length	Start category	Time period	End category 3	End category 4	End category 5	Total major	Combined total major
12 h	Tropical storm	Historical	0	0	0	0	Hist: 9
		Intermediate	0	0	0	0	
		Modern	0	0	0	0	Int: 18*
	Category 1	Historical	8	1	0	9	
		Intermediate	14*	3*	1*	18*	Mod: 28*
		Modern	20*	7*	1*	28*	
24 h	Tropical storm	Historical	2	1	0	3	Hist: 24
		Intermediate	7*	2*	0	9*	
		Modern	13*	4*	0	17*	Int: 39*
	Category 1	Historical	14	6	1	21	
		Intermediate	15	13*	2*	30*	Mod: 60*
		Modern	18	20*	5*	43*	
36 h	Tropical storm	Historical	12	2	0	14	Hist: 30
		Intermediate	16	7*	4*	27*	
		Modern	26*	17*	6*	49*	Int: 48*
	Category 1	Historical	3	9	4	16	
		Intermediate	6*	14*	1	21	Mod: 68*
		Modern	5	12	2	19	

The final column shows the combined totals of peak intensification events that result in storms evolving from either a tropical storm or Category 1 hurricane into a major hurricane for the historical era (Hist), the intermediate era (Int), and the modern era (Mod).

Values followed by an asterisk (*) indicate a total number of intensification events in the intermediate or modern era that would have had ≤ 5% chance of occurring in the historical era based on analyses using a binomial cumulative distribution function.

Analyses using a binomial cumulative distribution function reveal that increases in the number of intensification events that result in a TC transitioning from a weak TC into a major TC are statistically significant. Assuming historically observed rates of intensification events that cause TCs to transition from a weak TC into a major TC, it would be nearly impossible (0.01–0.04%) to observe the number of intensification events that result in TCs intensifying similarly in the intermediate era, and statistically impossible (0% chance) to observe the numbers of intensification events that result in TCs intensifying similarly in the modern era for all time windows. Assuming the observed rates of intensification events that cause TCs to transition from a weak TC into a major hurricane for the intermediate era, there is only a 2.1% chance of the total observed number of modern intensification events occurring for a 12-h window, and less than a 0.25% chance of the observed number of modern intensification events occurring for a 24-h window (0.22%) or 36-h window (0.20%).

### Variations in the locations where tropical cyclones intensify most quickly

**Figure 5:**
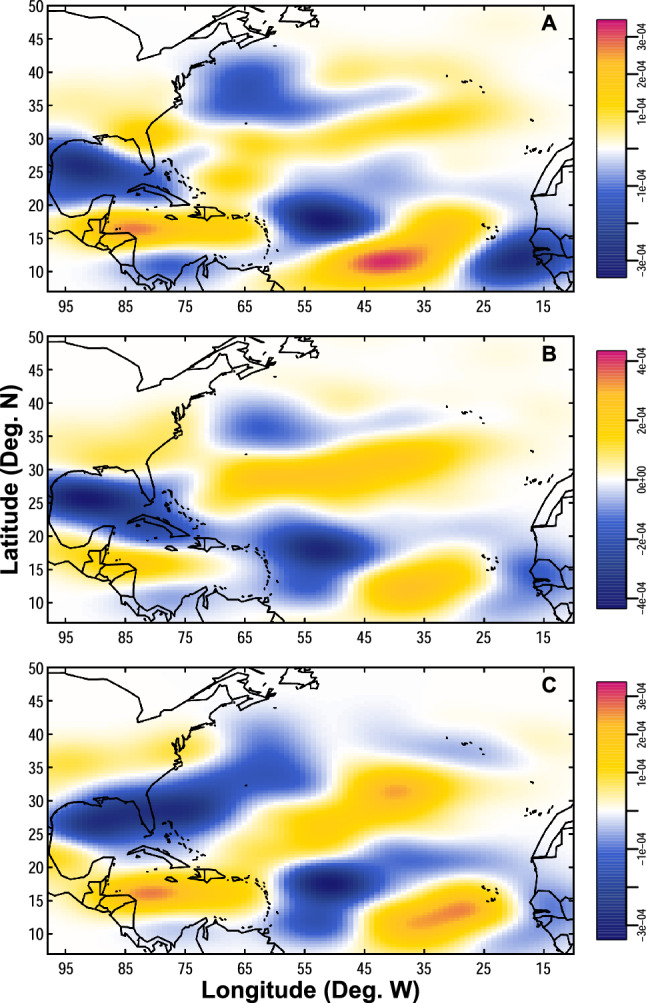
Density difference maps of the locations where tropical cyclones intensify most rapidly. Maps of the density differences in the locations where TCs intensify most quickly during (**A**) 12-h windows, (**B**) 24-h windows, and (**C**) 36-h windows. Differences are calculated for the modern era compared to the historical era by subtracting historical densities from modern densities. Units of density differences are track points per grid cell, where each map has 100 grid cells in both the latitudinal and longitudinal directions.

In addition to an amplification in overall peak intensification rates from the historical to the modern era, there are also variations in the locations at which TCs undergo their most rapid strengthening (Fig. 5, Fig. S2). From the historical to the modern era, there is a decrease in the likelihood of TCs intensifying most quickly in the Gulf of Mexico for all intensification windows (12-h, 24-h, and 36-h)—a finding that is consistent with other recent studies^28^. There is also a decrease in the likelihood of TCs intensifying most quickly in an area just east of the Caribbean Sea, though this decrease appears to be driven by a single slow-moving TC track in the historical era with multiple maximum intensification points that occurred in this region (Fig. S3). Alternatively, there are increases in the likelihood of TCs intensifying most quickly in the tropical eastern Atlantic, the southern Caribbean Sea, and in areas along and east of the U.S. Atlantic Coast for all time windows in the modern era compared to the historical era.

## Discussion

TCs are amongst costliest and most dangerous natural hazards to impact the U.S.^1–3^, and the hazard they present has grown as storms have become more extreme due to anthropogenic warming^4^. The rapid intensification of TCs in a warmer climate is particularly concerning, given that such events can be difficult to forecast and predict, leading to potentially escalated damages as well as difficulties when communicating the approaching hazard to coastal residents who may be in the TC’s path^28,4–39^. To help understand these challenges for our coastal communities, a variety of past work has focused on understanding how TC intensification rates may vary near U.S. coastlines or other specific regions of the Atlantic basin^22,27,28^, how the frequency of the most intense TCs may change in a warming world^1,40–42^, and connections between intensification and dissipation rates surrounding TC landfall events^32, 43^. Most of these studies have focused on particular subsets of TCs—for example, TCs that either meet certain intensification thresholds^27,30,44,45^ or intensify in specific regions^22,28,32^. Analyses presented here fill critical knowledge gaps by providing a broader assessment that focuses on overall changes to peak intensification rates for all observed Atlantic TCs from 1971 to 2020. Results show that peak TC intensification rates have already increased as anthropogenic emissions of greenhouse gases have warmed the planet, creating important implications for how the hazards our coastlines face may continue to evolve in a warming climate.

An analysis of maximum TC intensification rates during 12-, 24-, and 36-h windows over the past 50 years reveals that peak intensification rates of Atlantic TCs have significantly increased during this time. Although there are some differences in the shape of maximum intensification rate distributions across eras that should be further investigated in future work, mean maximum intensification rates increase steadily from the historical to the modern era, and are significantly greater (90% credible interval) in the modern era compared to the historical era (Fig. 1). While increases in mean TC intensification rates are not generally statistically significant from the intermediate era to the modern era, the monotonic progression across eras provides confidence in results that inevitably traverse times when different instruments and measurement techniques have been employed. Amplifications of rare but potentially highly damaging intensification events (99.5th percentile) are even more pronounced than increases in mean intensification rates (Figs. 1, 2), with increases from the historical era to the modern era of  >16 kts for 12- and 24-h windows, and >20 kts for a 36-h window (Fig. 2). These peak TC intensification rates have increased alongside anthropogenically-driven increases in SSTs, which have been rising at an increased rate in recent decades^18,42,46,47^. Sufficiently warm SSTs serve as a vital energy source for intensifying TCs^17,41,48,49^, and simultaneous increases in both extreme SSTs and maximum TC intensification rates suggests that human-caused warming has already had a measurable impact on the speed with which TCs strengthen.

Exceedance probabilities of TC intensification rates indicate that it has become statistically more likely in recent decades for TCs to strengthen at an accelerated pace (Fig. 3). The likelihood of TCs intensifying by 20 kts, 50 kts, or 65 kts within 12-, 24-, or 36-h tends to increase from the historical era to the modern era, and many of these increases are significant at a 90% credible interval (Fig. 3). These results are consistent with recent analyses that found statistically significant increases in global TC intensification rates of at least 50 kts in 24 h from 1990 to 2021^31^. Furthermore, there are similarly statistically significant increases from the historical to the modern era in the probability of TCs exceeding intensification rates of 40 kts within 24 h (Fig. S4), a threshold recently recommended as the definition for rapid intensification in the North Atlantic basin^50^. As a result of increasing intensification rates, there are decreases in the overall time required for TCs to strengthen by given amounts. For example, TCs that strengthen by at least 20 or 50 kts were about as likely (50 kts) or more likely (20 kts) to do so within 24 h in the intermediate and modern eras than to do so within 36 h in the historical era. These findings align well with previous studies that have suggested a shortening of the TC life cycle corresponding to changing intensification and weakening rates of TCs as the planet has warmed^34^.

Increased TC intensification rates have also resulted in a growing number of peak intensification events that result in TCs strengthening from weak TCs into major TCs in a short amount of time in the modern era compared to the historical or intermediate eras (Fig. 4). When considering each TC’s fastest intensification rate, the number of TC intensification events that result in a storm transitioning from a weak TC into a major TC more than triples from the historical to the modern era for a 12-h window, and more than doubles from the historical to the modern era for both 24- and 36-h windows (Fig. 4, Table 1). Analyses suggest that it would have been statistically impossible to achieve the total numbers of modern events that intensify from weak TCs to major TCs in any time window if the historical probabilities of such intensification had persisted through time. It is also statistically unlikely that modern totals of such TC intensification events would have been possible if the likelihood of such events occurring had not increased in the modern era compared to the intermediate era.

In the modern era, it becomes more common for TCs to intensify most quickly in the southern Caribbean Sea (east of central America), the central Atlantic (east of the U.S. Southeast coast), and the southeast Atlantic (off the west coast of Africa) compared to the historical era (Fig. 5, Fig. S2). Such spatial variations in peak TC intensification events have critical implications for coastlines around the Atlantic Basin. For example, TCs intensifying particularly quickly in the southwestern Caribbean Sea have the potential to create devastating impacts in many relatively resource-poor central American nations^51^. The vulnerability of these coastlines to such storms was exemplified by landfalls of particularly destructive TCs in this region in 2020, including Hurricane Eta^52^ and Hurricane Iota^53^, both of which quickly intensified into major hurricanes during the month of November as they approached the coast of Nicaragua^54^. Elsewhere, TCs that intensify at their fastest rate over the central part of the basin could be particularly dangerous for communities along the U.S. East coast—an area already threatened by other evolving TC hazards in a warming world, such as slower moving TCs and changing TC tracks^55–57^.

In a warming world, we expect changes to TC intensities, including the total number of very intense TCs^1,40–42^, or changes in the most extreme intensification rates^27,28,30,31^. Analyses presented here expand our understanding of changing TC intensification rates by performing a broad assessment of observed variations in overall maximum intensification rates for Atlantic TCs during the satellite era. Findings illustrate that it is possible to detect significant changes over the past 50 years to both the magnitude and locations of peak intensification rates of TCs in the Atlantic.

Historically, the most damaging TC events have been dominated by the most intense TCs to impact our coastlines^58^, and the vast majority of the most intense TCs undergo rapid intensification at some point in their lifecycle^45^. Because rapid intensification is often difficult to predict and forecast, quickly intensifying TCs can create communication and preparedness challenges for coastal communities in the storm’s path^26^. Results presented here suggest that the overall frequency and magnitude of quickly intensifying TC events have already increased for the Atlantic basin as the planet and our oceans have warmed over the past 50 years. Furthermore, the locations at which such intensification events occur have also varied. These changes may reasonably be expected to further amplify the substantial hazard and challenge that quickly intensifying TCs present to coastal communities. Findings presented here thus emphasize not only a need to curb greenhouse gas emissions and limit future warming, thereby limiting future increases to the rates at which TCs strengthen, but also a need for coastal planning and communication measures that will allow vulnerable communities to prepare for the changing hazard presented by quickly intensifying TC events.

## Methods

### HURDAT2 dataset

Data used for this analysis are from the open-access HURDAT2 database^35^, comprised of “best track” data from the National Hurricane Center, which is updated regularly to include new TC events and any changes to the historical record based on retrospective reviews of the data. The HURDAT2 database includes a variety of variables describing each TC at 6-h intervals along the TC track, including the date, time, key points along the TC track (such as landfall, minimum pressure, or maximum wind), status of the system, latitude, longitude, maximum sustained wind, minimum pressure, radii of 34-kt, 50-kt, and 64-kt winds, and the radius of maximum wind^35^. Analyses presented in this work primarily use the TC maximum wind speed, along with variables to describe when (date/time) and where (latitude/longitude) the maximum increases in wind speed occurred along the TC track.

Although the data contained within the HURDAT2 database begin in 1851, there are known discontinuities within TC records contained in the HURDAT2 database, including the introduction of aircraft measurements (1944) and the introduction of satellites (for which records were not available for the full Atlantic basin until the late 1960s)^35,59–62^. To avoid the impact of these two major discontinuities related to technological innovation and observation capabilities, analyses presented here focus only on records beginning in 1971 or later.

### Statistical analyses

Maximum intensification rates are measured by maximum increases in wind speeds for each Atlantic TC from 1971 to 2020 across 12-h, 24-h, and 36-h windows. These maximum changes in wind speed are found by calculating wind speed differences at each 6-h time step along the TC track where possible. Equation (1) describes this process:

1 $${\Delta MaxWind}_{i}= {MaxWind}_{{t}_{i+ \frac{W}{6}} }- {MaxWind}_{{t}_{i}},$$

where $MaxWind$ represents the maximum wind speed recorded in the HURDAT2 database, $i$ represents each point along the TC track, $t$ represents the time step, and $W$ represents the window length (12, 24, or 36 h). If the TC track is not long enough to calculate the difference, the magnitude of maximum wind increase for that TC track/time window is simply listed as NA, and not included in full analyses. For example, if a TC track lasted only 30 h, and thus had only five 6-hourly timesteps, it would not be possible to calculate a 36-h maximum wind speed change, so that track would not be included in analyses of 36-h maximum intensification rates. Because of this approach, time windows of 12 h typically have the most datapoints, and time windows of 36 h typically have the fewest datapoints, though no time window has fewer than 290 datapoints in any era.

If multiple time steps along a TC track have a change in wind speed that equates to the maximum intensification rate for a given time window, the magnitude of the wind speed change is only included once for analyses that focus on overall distributional changes (e.g., Figs. 1, 2, 3). However, the starting and ending TC category for each maximum wind increase are retained for pre-intensification and post-intensification Saffir–Simpson category heatmaps (Fig. 4), and all locations at which the maximum wind increase occurs are retained for analyses of variations in the peak intensification location (Fig. 5). For example, if a TC in the historical era had a maximum wind increase of 30 kts across a 12-h window, but that maximum wind increase occurred at four different 12-h windows during the TC’s lifetime, the 30 kt wind increase would only be counted once for analyses of peak TC intensification rate distributions (Figs. 1, 2, 3). However, the pre-intensification and post-intensification Saffir–Simpson category of the TC for all four wind increases would be included in analyses for Fig. 4 (since pre- and post-intensification categories are likely to vary across the four intensification events), and all four locations at which the TC experienced a 30-kt wind increase would be retained for analyses of changing intensification locations in Fig. 5. Locations at which TCs experience the greatest change in intensification rates are calculated as the mid-point between the latitude/longitude at the start of the time window, and the latitude/longitude at the end of the time window.

It is worth noting that although maximum wind speeds are provided at 5-kt intervals within the HURDAT2 best track data, some results presented here are assessed as significant even when changes from one era to another are less than 5 kts. For instance, there is a significant increase in the mean maximum intensification rates for a 24-h window from the historical (17.7 kts) to the modern era (22.5 kts) of 4.8 kts (Fig. 1). Determining significance in such cases is possible by assessing credible intervals that are constructed from 10,000 bootstrapped samples of the original dataset—an approach that is used for both distribution means (Fig. 1) and exceedance probabilities (Fig. 3). Bootstrapping is a statistical technique in which a dataset is resampled *n* times (here, *n* = 10,000) with replacement to generate *n* new sample datasets that are equal in length to the original^63,64^. For Fig. 1, 10,000 bootstrap samples of the mean of the maximum intensification rate distributions are generated to determine the 90% CI (p = 0.1) of the mean in each era. To assess the likelihood of TC maximum intensification rates exceeding either 20-kts, 50-kts, or 65-kts over various time windows, 10,000 bootstrap samples are generated to construct a 90% CI (p = 0.1) of the exceedance probability in each era (Fig. 3). If the bounds of the credible intervals for two statistics (for instance, the mean) in different eras do not overlap, the result is considered significant for that credible interval, even if the magnitude of the difference between time periods is less than the 5-kt interval used for maximum wind speed in the HURDAT2 dataset.

Heatmaps are used to visualize variations in the pre-intensification and post-intensification Saffir–Simpson category of TCs from the historical to the modern era (Fig. 4). These heatmaps are created by first constructing a heatmap to illustrate the distribution of pre-intensification and post-intensification TC categories for all intensification events over a particular time window in a particular era (e.g., Fig. S1). After constructing these heatmaps for all eras/time windows, the historical (Fig. S1a,d,g) and intermediate era (Fig. S1b,e,h) heatmaps are subtracted from the modern heatmaps (Fig. S1c,f,i) for each time window. The resulting heatmaps (Fig. 4) illustrate differences in pre-intensification and post-intensification Saffir–Simpson category across different eras for each time window. Because the focus of this analysis is upon intensification of TCs, heatmap boxes that would indicate weakening (i.e., decreases in TC category) are grayed out in Fig. 4 and Fig. S1. Nevertheless, the question of how TC weakening rates may change in a warming climate is important, and would be worth pursuing in future research^34,65^.

Density difference maps showing spatial shifts in the locations at which TCs intensify most quickly are generated using a kernel density estimate in both the modern and historical eras (Fig. S2), and then subtracting the historical estimate from the modern estimate (Fig. 5). This approach follows that used in Refs.^55,57^. Units of densities shown on the maps in Fig. S2 and Fig. 5 are track points per grid cell, where each map has 100 grid cells in both the latitudinal and longitudinal directions. The normal reference rule is used to calculate the bandwidth for the kernel density estimation, an approach to choosing a bandwidth that has been demonstrated to work exceptionally well for a broad set of use cases^66^.

### Supplementary Information

Supplementary Figures.

## Data Availability

The Best Track Datasets analyzed during the current study are available in the Atlantic Hurricane Database (HURDAT2) at https://www.nhc.noaa.gov/data/hurdat/hurdat2-1851-2020-052921.txt.

## References

[1] Emanuel K (2005). Increasing destructiveness of tropical cyclones over the past 30 years. Nature.

[2] Pielke RA (2007). Future economic damage from tropical cyclones: Sensitivities to societal and climate changes. Philos. Trans. R. Soc. A Math. Phys. Eng. Sci..

[3] Rappaport EN (2014). Fatalities in the United States from Atlantic tropical cyclones: New data and interpretation. Bull. Am. Meteorol. Soc..

[4] Grinsted A, Ditlevsen P, Christensen JH (2019). Normalized US hurricane damage estimates using area of total destruction, 1900–2018. Proc. Natl. Acad. Sci..

[5] NOAA National Centers for Environmental Information (NCEI). *U.S. Billion-Dollar Weather and Climate Disasters*. https://www.ncdc.noaa.gov/billions/events/US/1980-2020 (2023).

[6] Pasch, R. J., Penny, A. B. & Berg, R. *Hurricane Maria*. *National Hurricane Center Tropical Cyclone Report* (2023).

[7] Simpson RH, Saffir H (1974). The hurricane disaster potential scale. Weatherwise.

[8] Blake, E. S. & Zelinsky, D. A. *National Hurricane Center Tropical Cyclone Report: Hurricane Harvey* (2017).

[9] NOAA National Centers for Environmental Information (NCEI). *Monthly National Climate Report for September 2022* (2022).

[10] Blake, E. S., Kimberlain, T. B., Berg, R. J., Cangialosi, J. P. & Beven, J. L. *Tropical Cyclone Report: Hurricane Sandy (AL182012) 22–29 October 2012* (2013).

[11] Beven, J. L., Hagen, A. & Berg, R. *National Hurricane Center Tropical Cyclone Report: Hurricane Ida (AL092021)*. *National Huricane Center Tropical Cyclone Report* (2022).

[12] Cangialosi, J. P., Latto, A. S. & Berg, R. *National Hurricane Center Tropical Cyclone Report: Hurricane Irma (AL112017)* (2021).

[13] Kaplan J, DeMaria M, Kaplan J, DeMaria M (2003). Large-scale characteristics of rapidly intensifying tropical cyclones in the North Atlantic basin. Weather Forecast..

[14] Song J, Duan Y, Klotzbach PJ (2020). Increasing trend in rapid intensification magnitude of tropical cyclones over the western North Pacific. Environ. Res. Lett..

[15] Lin II, Chen CH, Pun IF, Liu WT, Wu CC (2009). Warm ocean anomaly, air sea fluxes, and the rapid intensification of tropical cyclone Nargis (2008). Geophys. Res. Lett..

[16] Sun J, Zuo J, Ling Z, Yan Y (2016). Role of ocean upper layer warm water in the rapid intensification of tropical cyclones: A case study of typhoon Rammasun (1409). Acta Oceanol. Sin..

[17] Emanuel K (1987). The dependence of hurricane intensity on climate. Nature.

[18] Fox-Kemper B, Masson-Delmotte V (2021). Ocean, cryosphere and sea level change. Climate Change 2021: The Physical Science Basis. Contribution of Working Group I to the Sixth Assessment Report of the Intergovernmental Panel on Climate Change.

[19] Knutson T (2019). Tropical cyclones and climate change assessment: Part II. Projected response to anthropogenic warming. Bull. Am. Meteorol. Soc..

[20] Villarini G, Vecchi GA (2013). Projected increases in North Atlantic tropical cyclone intensity from CMIP5 models. J. Clim..

[21] Knutson TR (2015). Global projections of intense tropical cyclone activity for the late twenty-first century from dynamical downscaling of CMIP5/RCP4.5 scenarios. J. Clim..

[22] Kossin JP (2017). Hurricane intensification along United States coast suppressed during active hurricane periods. Nature.

[23] Hall TM, Kossin JP, Thompson T, McMahon J (2021). U.S. tropical cyclone activity in the 2030s based on projected changes in tropical sea surface temperature. J. Clim..

[24] Seneviratne SI, Masson-Delmotte V (2021). Weather and climate extreme events in a changing climate. Climate Change 2021: The Physical Science Basis. Contribution of Working Group I to the Sixth Assessment Report of the Intergovernmental Panel on Climate Change.

[25] Trabing BC, Bell MM (2020). Understanding error distributions of hurricane intensity forecasts during rapid intensity changes. Weather Forecast..

[26] DeMaria M, Franklin JL, Onderlinde MJ, Kaplan J (2021). Operational forecasting of tropical cyclone rapid intensification at the National Hurricane Center. Atmosphere.

[27] Balaguru K, Foltz GR, Leung LR (2018). Increasing magnitude of hurricane rapid intensification in the Central and Eastern tropical Atlantic. Geophys. Res. Lett..

[28] Balaguru K (2022). Increasing hurricane intensification rate near the US Atlantic coast. Geophys. Res. Lett..

[29] Wang C, Wang X, Weisberg RH, Black ML (2017). Variability of tropical cyclone rapid intensification in the North Atlantic and its relationship with climate variations. Clim. Dyn..

[30] Bhatia KT (2019). Recent increases in tropical cyclone intensification rates. Nat. Commun..

[31] Klotzbach PJ (2022). Trends in global tropical cyclone activity: 1990–2021. Geophys. Res. Lett..

[32] Zhu YJ, Collins JM, Klotzbach PJ (2021). Nearshore hurricane intensity change and post-landfall dissipation along the United States Gulf and East Coasts. Geophys. Res. Lett..

[33] Mei W, Xie SP (2016). Intensification of landfalling typhoons over the northwest Pacific since the late 1970s. Nat. Geosci..

[34] Wang S, Rashid T, Throp H, Toumi R (2020). A shortening of the life cycle of major tropical cyclones. Geophys. Res. Lett..

[35] Landsea CW, Franklin JL (2013). Atlantic hurricane database uncertainty and presentation of a new database format. Mon. Weather Rev..

[36] Sapp JW, Alsweiss SO, Jelenak Z, Chang PS, Carswell J (2019). Stepped frequency microwave radiometer wind-speed retrieval improvements. Remote Sens..

[37] Emanuel K (2017). Will global warming make hurricane forecasting more difficult?. Bull. Am. Meteorol. Soc..

[38] Tierra MCM, Bagtasa G (2023). Identifying the rapid intensification of tropical cyclones using the Himawari-8 satellite and their impacts in the Philippines. Int. J. Climatol..

[39] Yang J, Chen M (2021). Variations of rapidly intensifying tropical cyclones and their landfalls in the Western North Pacific. Coast. Eng. J..

[40] Bender MA (2010). Modeled impact of anthropogenic warming on the frequency of intense Atlantic hurricanes. Science.

[41] Elsner JB, Kossin JP, Jagger TH (2008). The increasing intensity of the strongest tropical cyclones. Nature.

[42] Kossin JP, Knapp KR, Olander TL, Velden CS (2020). Global increase in major tropical cyclone exceedance probability over the past four decades. Proc. Natl. Acad. Sci. U.S.A..

[43] Li L, Chakraborty P (2020). Slower decay of landfalling hurricanes in a warming world. Nature.

[44] Kaplan J, Demaria M, Knaff JA (2010). A revised tropical cyclone rapid intensification index for the Atlantic and Eastern North Pacific basins. Weather Forecast..

[45] Lee C-Y, Tippett MK, Sobel AH, Camargo SJ (2016). Rapid intensification and the bimodal distribution of tropical cyclone intensity. Nat. Commun..

[46] Santer BD (2006). Forced and unforced ocean temperature changes in Atlantic and Pacific tropical cyclogenesis regions. Proc. Natl. Acad. Sci. U.S.A..

[47] Gillett NP, Stott PA, Santer BD (2008). Attribution of cyclogenesis region sea surface temperature change to anthropogenic influence. Geophys. Res. Lett..

[48] Gray, W. M. *Tropical Cyclone Genesis* (1975).

[49] Strazzo SE, Elsner JB, LaRow TE (2015). Quantifying the sensitivity of maximum, limiting, and potential tropical cyclone intensity to SST: Observations versus the FSU/COAPS global climate model. J. Adv. Model. Earth Syst..

[50] Li Y, Tang Y, Toumi R, Wang S (2022). Revisiting the definition of rapid intensification of tropical cyclones by clustering the initial intensity and inner-core size. J. Geophys. Res. Atmos..

[51] Ishizawa OA, Miranda JJ (2019). Weathering storms: Understanding the impact of natural disasters in Central America. Environ. Resour. Econ. (Dordr.).

[52] Pasch, R. J., Reinhart, B. J., Berg, R. & Roberts, D. P. *National Hurricane Center Tropical Cyclone Report: Hurricane Eta (AL292020)* (2021).

[53] Stewart, S. R. *National Hurricane Center Tropical Cyclone Report: Hurricane Iota (AL312020)* (2021).

[54] Klotzbach PJ (2022). A hyperactive end to the Atlantic hurricane season October–November 2020. Bull. Am. Meteorol. Soc..

[55] Garner AJ, Kopp RE, Horton BP (2021). Evolving tropical cyclone tracks in the North Atlantic in a warming climate. Earths Future.

[56] Hall TM, Kossin JP (2019). Hurricane stalling along the North American coast and implications for rainfall. NPJ Clim. Atmos. Sci..

[57] Weaver MM, Garner AJ (2023). Varying genesis and landfall locations for North Atlantic tropical cyclones in a warmer climate. Sci. Rep..

[58] Grinsted A, Ditlevsen P, Christensen JH (2019). Normalized US hurricane damage estimates using area of total destruction, 1900–2018. Proc. Natl. Acad. Sci. U.S.A..

[59] Vecchi GA, Knutson TR (2008). On estimates of historical North Atlantic tropical cyclone activity. J. Clim..

[60] Vecchi GA, Knutson TR (2011). Estimating annual numbers of Atlantic hurricanes missing from the HURDAT database (1878–1965) using ship track density. J. Clim..

[61] Chang EKM, Guo Y (2007). Is the number of North Atlantic tropical cyclones significantly underestimated prior to the availability of satellite observations?. Geophys. Res. Lett..

[62] Mann ME, Sabbatelli TA, Neu U (2007). Evidence for a modest undercount bias in early historical Atlantic tropical cyclone counts. Geophys. Res. Lett..

[63] Efron B (1979). Bootstrap methods: Another look at the jackknife. Ann. Stat..

[64] Efron B, Tibshirani RJ (1993). An Introduction to the Bootstrap.

[65] Wood KM, Ritchie EA (2015). A definition for rapid weakening of North Atlantic and eastern North Pacific tropical cyclones. Geophys. Res. Lett..

[66] Sheather SJ, Jones MC (1991). A reliable data-based bandwidth selection method for Kernel density estimation. J. R. Stat. Soc. Ser. B (Methodol.).

