# Supplementary material for: Observed increases in North Atlantic tropical cyclone peak intensification rates
Source: Sci Rep. 2023 Oct 19;13:16299. doi: 10.1038/s41598-023-42669-y (PMC10587146; doi:10.1038/s41598-023-42669-y)
Supplement: Supplementary file 1 — Supplementary Figures. [file 41598_2023_42669_MOESM1_ESM.docx]

# Supplementary Material: Observed Increases in North Atlantic Tropical Cyclone Peak Intensification Rates


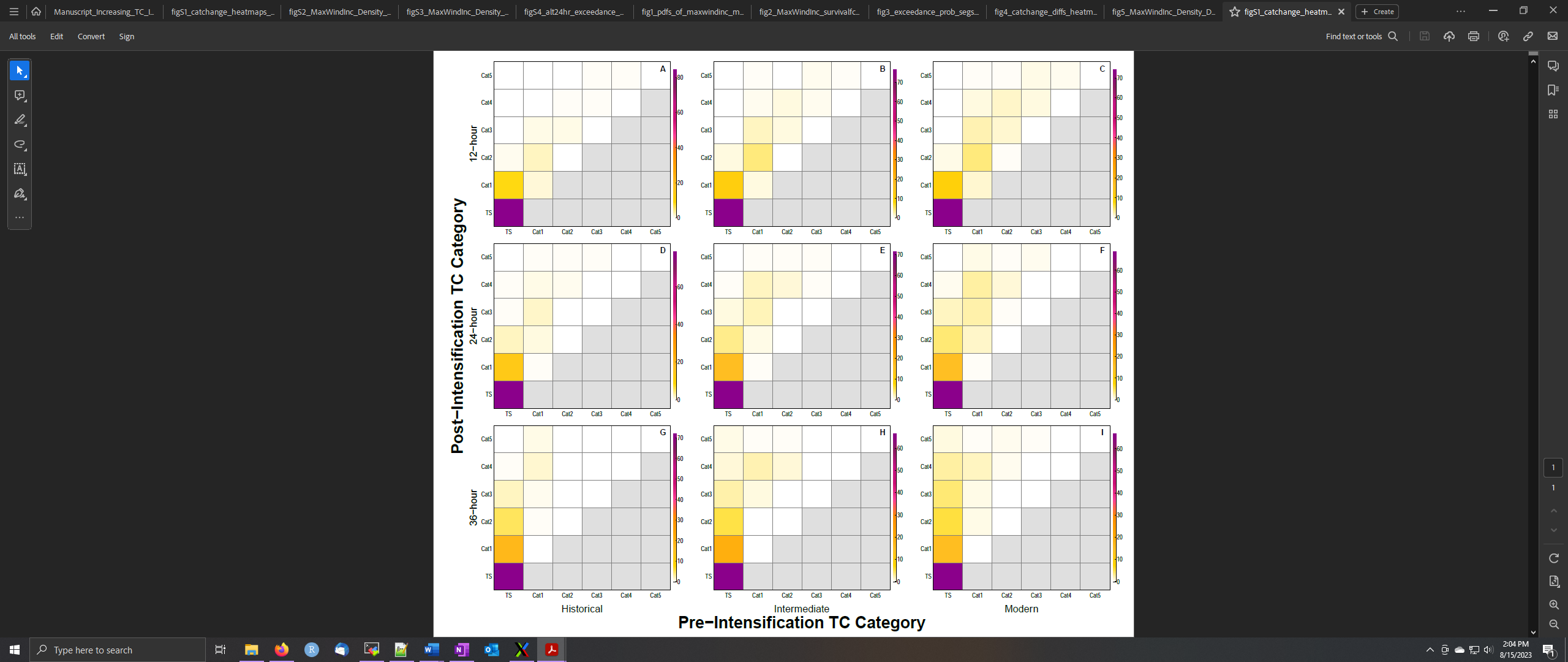


**Figure S1—Heatmaps of Tropical Cyclone Saffir-Simpson Category Changes |** Heatmaps showing percentages of TCs that fall into specific pre- and post-intensification TC categories in the historical era (a, d, g), the intermediate era (b, e, h), and the modern era (c, f, g). Results are shown for peak intensification events across a-c) 12-hour windows, d-f) 24-hour windows, and g-i) 36-hour windows. Units for color bars are percentages. Note that because the focus of the analyses presented here is on intensification, blocks that would indicate weakening are gray.


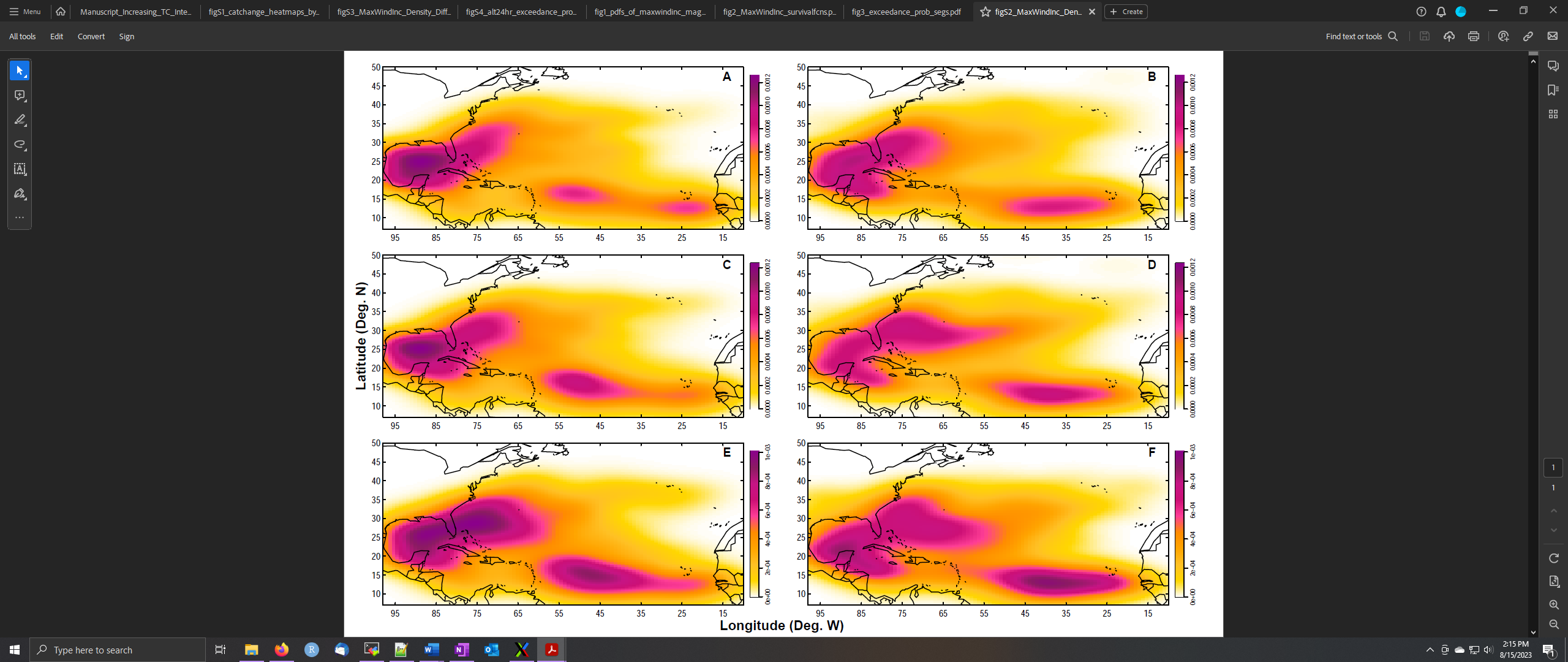


**Figure S2--Density Maps of the Locations Where Tropical Cyclones Intensify Most Rapidly |** Maps of the density of the locations where TCs intensify most quickly during a-b) 12-hour windows, c-d) 24-hour windows, and e-f) 36-hour windows. Differences are calculated for the historical era (a, c, e) and the modern era (b, d, f). Units of density differences are track points per grid cell, where each map has 100 grid cells in both the latitudinal and longitudinal directions.


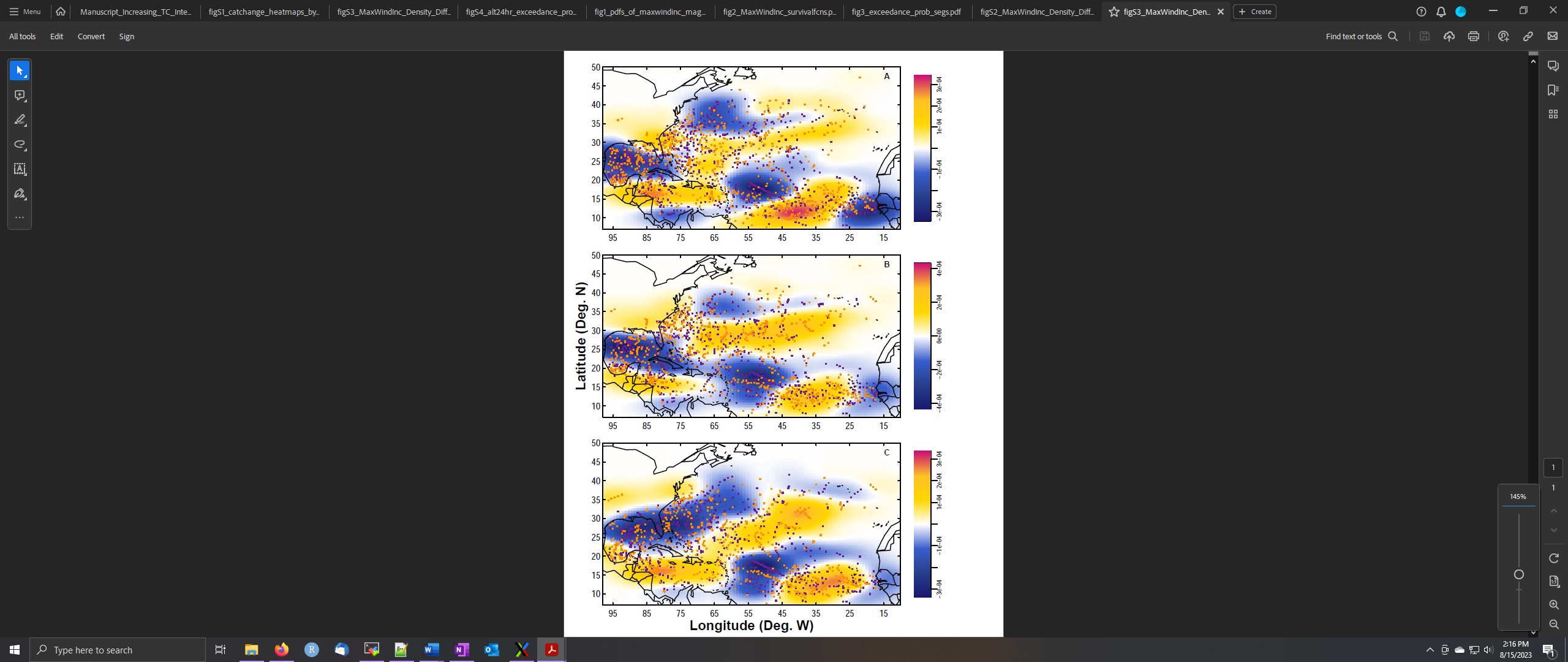


**Figure S3--Density Difference Maps and Scatter Plots of the Locations Where Tropical Cyclones Intensify Most Rapidly |** Maps of the density differences in the locations where TCs intensify most quickly during a) 12-hour windows, b) 24-hour windows, and c) 36-hour windows. Differences are calculated for the modern era compared to the historical era by subtracting historical densities from modern densities. Units of density differences are track points per grid cell, where each map has 100 grid cells in both the latitudinal and longitudinal directions. Overlaid on density differences are points to show the locations where TCs intensify most quickly in the modern era (orange) compared to the historical era (purple).


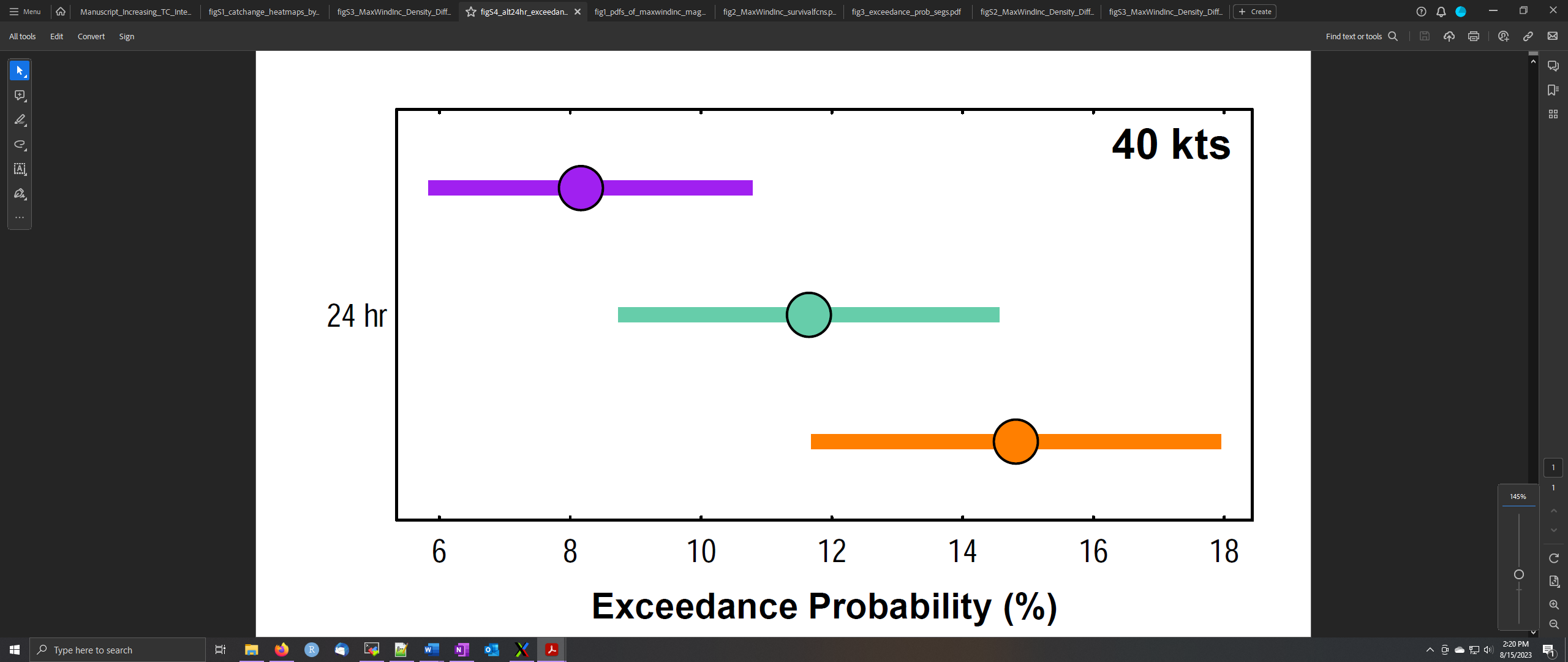


**Figure S4—40-kt Exceedance Probabilities of Tropical Cyclone Maximum 24-hr Intensification Rates | Probabilities of TCs exceeding an intensification rate of 40 kts during a 24-hour window for the historical era (purple), intermediate era (teal), and modern era (orange). Circles represent the observed exceedance probabilities; colored segments show the bootstrapped 90% credible interval of the observed exceedance probability.**
